# Rapid Effects of Hearing Song on Catecholaminergic Activity in the Songbird Auditory Pathway

**DOI:** 10.1371/journal.pone.0039388

**Published:** 2012-06-19

**Authors:** Lisa L. Matragrano, Michaël Beaulieu, Jessica O. Phillip, Ali I. Rae, Sara E. Sanford, Keith W. Sockman, Donna L. Maney

**Affiliations:** 1 Department of Psychology, Emory University, Atlanta, Georgia, United States of America; 2 Department of Biology, University of North Carolina, Chapel Hill, North Carolina, United States of America; 3 Curriculum in Neurobiology, University of North Carolina, Chapel Hill, North Carolina, United States of America; Claremont Colleges, United States of America

## Abstract

Catecholaminergic (CA) neurons innervate sensory areas and affect the processing of sensory signals. For example, in birds, CA fibers innervate the auditory pathway at each level, including the midbrain, thalamus, and forebrain. We have shown previously that in female European starlings, CA activity in the auditory forebrain can be enhanced by exposure to attractive male song for one week. It is not known, however, whether hearing song can initiate that activity more rapidly. Here, we exposed estrogen-primed, female white-throated sparrows to conspecific male song and looked for evidence of rapid synthesis of catecholamines in auditory areas. In one hemisphere of the brain, we used immunohistochemistry to detect the phosphorylation of tyrosine hydroxylase (TH), a rate-limiting enzyme in the CA synthetic pathway. We found that immunoreactivity for TH phosphorylated at serine 40 increased dramatically in the auditory forebrain, but not the auditory thalamus and midbrain, after 15 min of song exposure. In the other hemisphere, we used high pressure liquid chromatography to measure catecholamines and their metabolites. We found that two dopamine metabolites, dihydroxyphenylacetic acid and homovanillic acid, increased in the auditory forebrain but not the auditory midbrain after 30 min of exposure to conspecific song. Our results are consistent with the hypothesis that exposure to a behaviorally relevant auditory stimulus rapidly induces CA activity, which may play a role in auditory responses.

## Introduction

The processes of attending to a stimulus and assigning value to it both depend on catecholamine neuromodulators such as dopamine (DA) and norepinephrine (NE). Catecholaminergic (CA) systems can alter sensory gating and receptive fields to maximize the salience of behaviorally relevant signals. In so doing, they serve as dynamic filters that integrate prior experience, environmental context, and internal state [1], [2]. When the importance of an auditory stimulus is increased, for example by associating it with a foot shock or a reward, the resulting remapping of auditory cortex is accomplished in part by dopaminergic neuromodulation [3]. Similarly, because CA systems are exquisitely sensitive to the animal’s environment, they can bring information on context directly to areas involved in sensory processing to facilitate context-appropriate responses to sensory signals [2], [4].

In songbirds, CA projections to the auditory forebrain have been hypothesized to affect the processing of song [5]–[13]. Noradrenergic denervation or blockade of CA activity reduces behavioral and neural responses to song as well as behavioral and neural selectivity for sexually stimulating song [14]–[18]. In female white-throated sparrows (*Zonotrichia albicollis*), sexual receptivity is associated with an increase in the number of CA neurons in brainstem cell groups likely to project to auditory areas [10], as well as denser CA innervation of the auditory forebrain and midbrain [10], [19]. Female European starlings (*Sturnus vulgaris*) exposed for one week to high-quality male song have a greater density of CA fibers in the auditory forebrain than females exposed to low-quality song [20]. Thus, there is evidence that catecholaminergic projections to auditory areas may carry information on internal state and social context, that these projections regulate behavioral and neural responses to song, and that hearing song may itself alter catecholamine levels in the auditory system.

Despite continued interest in the role of catecholamines in the processing of song [13], the mechanisms by which they modulate auditory responses in songbirds are not well understood. According to models developed in mammals, CA terminals may release transmitter in a tonic or paracrine fashion that is independent of both firing and external stimuli [21]. This release may alter the responsivity or spontaneous firing activity in forebrain neurons [22] and may be sustained over prolonged periods [23]. Such release may drive sensory plasticity without stimulus-dependent firing or presynaptic regulation. In contrast, or in addition, catecholamine release may be driven by sensory stimuli. Neurons in both the NE and DA systems respond to a myriad of stimuli from multiple modalities [1], [4], [24], [25]. Hearing conspecific song induces the expression of immediate early genes such as FOS and Egr-1, which mark new protein synthesis in response to a stimulus [26], in many CA regions. In male zebra finches (*Taenopygia guttata*), for example, exposure to a singing tutor induced FOS expression in the substantia nigra (SN), ventral tegmental area (VTA), and the periaqueductal gray (PAG), but this induction was found within DA neurons only in the PAG [27]. Similarly, in estrogen-primed female white-throated sparrows, hearing conspecific male song induced Egr-1 expression in the locus coeruleus (LoC), SN, VTA, and PAG [28, unpublished data], but not in the tyrosine hydroxylase (TH)-positive cells of those areas [10]. Gale and Perkel [29] showed that in anesthetized male zebra finches, dopaminergic neurons in the SN and VTA fired in response to auditory stimuli, including conspecific song. Such responses may be limited, however, to the anesthetized state [30], [31]. It is therefore unclear whether hearing conspecific song rapidly induces CA activity in awake individuals. In this study, we looked for evidence of rapid increases in catecholamine synthesis in auditory areas of sexually receptive females listening to conspecific male song.

In order to test whether hearing song rapidly engages CA inputs to auditory areas, we employed a relatively new immunohistochemical method to quantify synthetic activity of TH. We took advantage of the fact that in order to synthesize catecholamines, TH must be phosphorylated at a minimum of one of its four serine sites. Immunolabeling of phosphorylated TH (pTH) can thus be used to map active catecholamine synthesis [32]. Dopamine synthesis and release are most highly correlated with phosphorylation at serine site 40 (ser40) [33], [34], which is not phosphorylated at high rates in the resting state [32], [35]. The phosphorylation of TH at ser40 in response to stimuli is quite rapid; administration of pharmacological agents such as haloperidol or acetylcholine induces phosphorylation within minutes both *in vivo* and *in vitro* [36]. Antibodies directed against pTH(ser40) have been used to examine the effects of social stimuli on TH activity in the brain [37], [38]. Here, we hypothesized that exposure to male conspecific song would increase immunoreactivity for pTH(ser40), referred to hereafter as pTH, in the auditory pathway of females within minutes. In order to confirm that we were able to detect changes in CA activity *via* immunohistochemistry (IHC), we also used HPLC to more directly quantify the concentrations of catecholamines and their metabolites in auditory areas.

**Figure 1:**
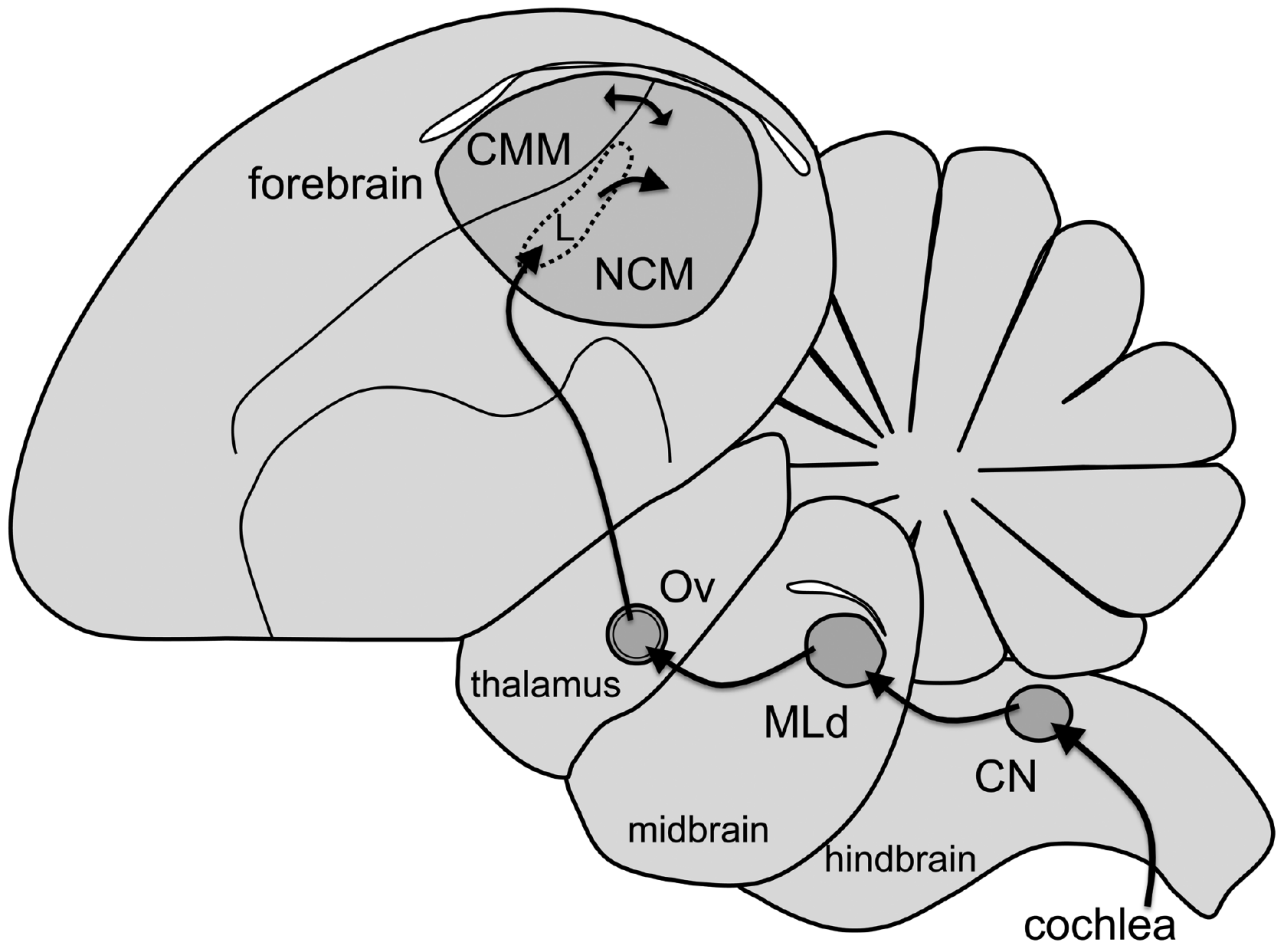
Parasagittal view of the auditory pathway in songbirds. Rostral is to the left. The auditory nerve enters the brainstem and projects to the cochlear nucleus (CN). From CN, projections extend to the auditory midbrain (MLd, the dorsal lateral mesencephalic nucleus). MLd projects to the auditory thalamus (Ov, nucleus ovoidalis), which projects to the thalamorecipient region of the auditory forebrain, Field L. From Field L, projections extend to the caudomedial nidopallium (NCM). NCM is reciprocally connected to the caudomedial mesopallium (CMM).

The anatomical and functional organization of the central auditory pathway in songbirds largely resembles that found in other vertebrates, including mammals (Fig. 1) [39]. Auditory input is transduced in the cochlea, ascends through brainstem areas analogous to mammalian cochlear nuclei, and arrives at the dorsal lateral mesencephalic nucleus (MLd), the avian homolog of the mammalian inferior colliculus, or auditory midbrain [40]. MLd neurons send direct projections to the thalamic nucleus Ovoidalis (Ov), the avian homolog of the ventral medial geniculate [40]. Both structures participate in auditory discrimination and are tuned to behaviorally relevant signals [39], [41], [42]. Ov projects to a pronounced lobe in the forebrain that contains auditory areas (Fig. 1). Inside this lobe, the caudomedial nidopallium (NCM) receives input from the thalamo-recipient Field L and is heavily interconnected with the caudomedial mesopallium (CMM). NCM and CMM are analogous to the supragranular layers of mammalian auditory cortex [43] or to mammalian auditory association cortex [44], [45]. In this study, we quantified sound-induced CA activity both immunohistochemically and *via* HPLC at multiple levels of this pathway [19], [46].

## Results

### Rapid Effects of Song on TH Phosphorylation

**Figure 2:**
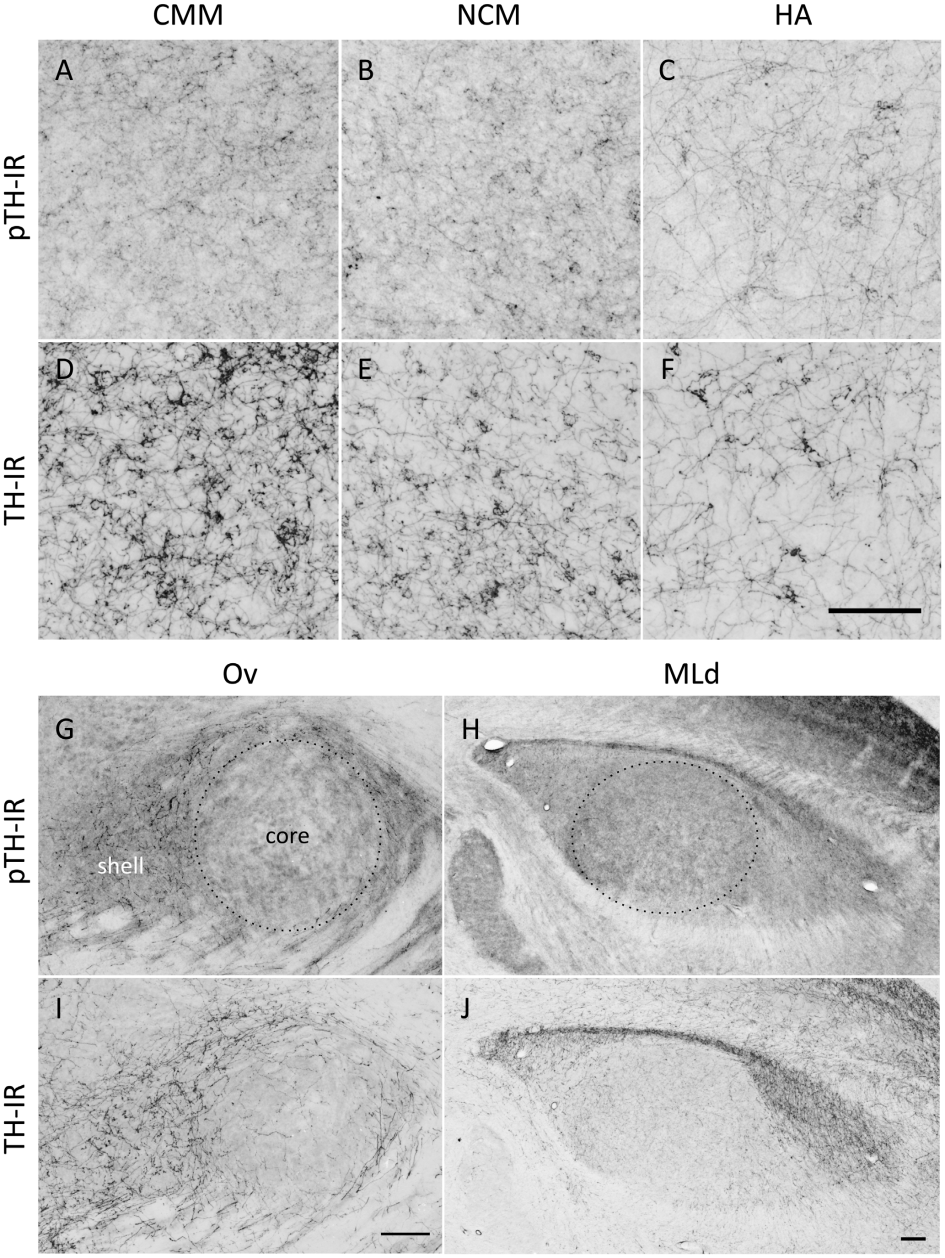
Examples of immunoreactive fibers labeled in this study. Immunoreactivity (IR) for phosphorylated tyrosine hydroxylase (pTH; A, B, C, G, H) or tyrosine hydroxylase (TH; D, E, F, I, J) is shown in the caudomedial mesopallium (CMM; A, D), caudomedial nidopallium (NCM; B, E), apical hyperpallium (HA; C, F), n. Ovoidalis (Ov; G, I) and the dorsal lateral mesencephalic nucleus (MLd; H, J) in birds that heard 15 min of song. Dotted lines encircle the areas sampled in the Ov core and MLd. Rostral is to the right. Scale bars, 100 µm.

**Figure 3:**
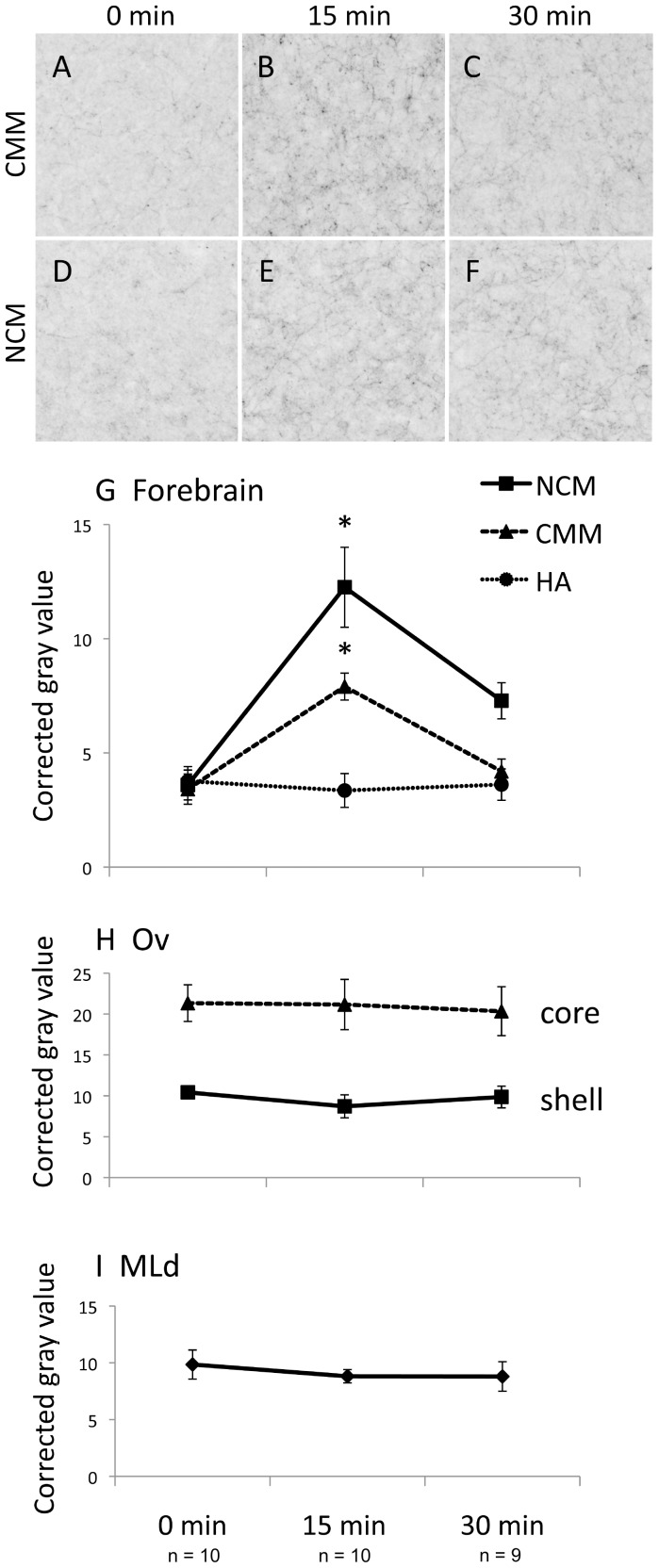
The effects of song exposure on phosphorylated tyrosine hydroxylase immunoreactivity (pTH-IR). A–F. Immunoreactive fibers in the auditory forebrain in a typical bird (median corrected gray value) from each group. G. pTH-IR increased in CMM and NCM after 15 min of song, and in NCM remained elevated above baseline after 30 min. H, I. pTH-IR did not increase in Ov or MLd. NCM, caudomedial nidopallium. CMM, caudomedial mesopallium. HA, apical hyperpallium. Ov, n. Ovoidalis (auditory thalamus). MLd, dorsal portion of the lateral mesencephalic nucleus (auditory midbrain). *significantly different from the silence (0 min song) condition, see text for p values.

We exposed individually housed, E2-treated female white-throated sparrows to audio recordings of conspecific male song, collected brain tissue immediately afterwards, and quantified the phosphorylation of TH in the auditory pathway *via* IHC. The auditory forebrain (NCM and CMM), thalamus (Ov) and midbrain (MLd) showed robust immunoreactivity for pTH and total TH (Fig. 2). Fifteen min of song exposure caused an increase in pTH immunoreactivity in the auditory forebrain, indicating the rapid engagement of catecholaminergic synthetic machinery (Fig. 3). pTH immunoreactivity increased in both NCM and CMM after 15 min of song exposure (NCM: z = 5.20, P<0.001; CMM: z = 5.35, P<0.001) and after 30 min of song exposure was still elevated above baseline in NCM (z = 2.16, P = 0.031). By contrast, we did not detect an effect of song exposure in the auditory thalamus or midbrain (|z| ≤1.03, P≥0.304). Our finding of song-induced TH phosphorylation was thus limited to the auditory forebrain. To help assess whether this effect was specific to the auditory system we also looked at a visual area in the forebrain, the apical part of the hyperpallium (HA), and found no effect of song duration (|z| ≤0.43, P≥0.669).

**Figure 4:**
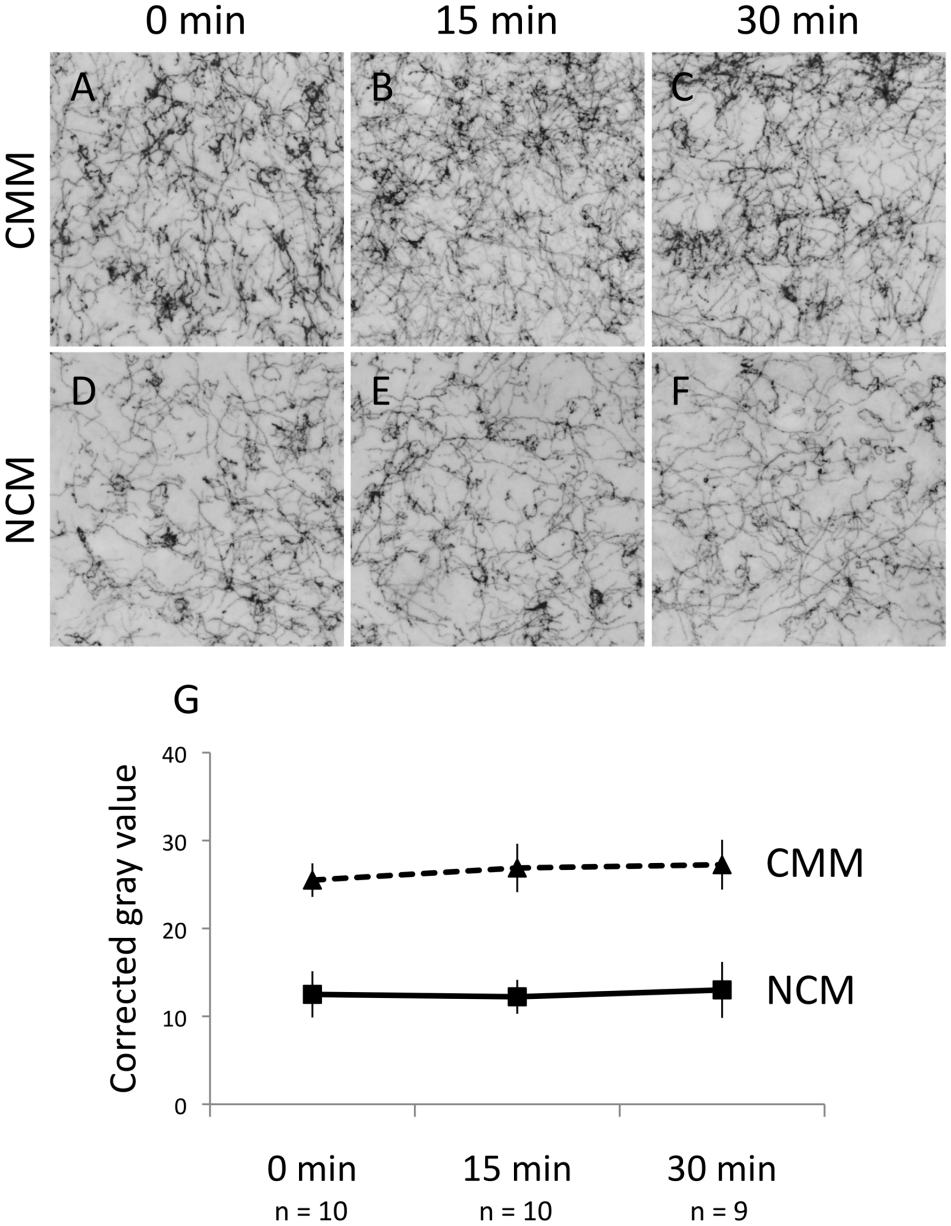
There were no effects of song exposure on tyrosine hydroxylase immunoreactivity (TH-IR). A–F. TH-immunoreactive fibers in the auditory forebrain in a typical bird (median corrected gray value) from each group. G. TH-IR remained unchanged in CMM and NCM after 15 min and 30 min of song. CMM, caudomedial mesopallium. NCM, caudomedial nidopallium.

We found no significant effects of song duration on TH immunoreactivity (|z| ≤1.51, P≥0.13; Fig. 4), which indicates that hearing song did not alter the availability or synthesis of TH itself. Rather, the available TH was more likely to become phosphorylated, and therefore active, in response to song exposure.

### Rapid Effects of Song on Catecholamines and their Metabolites

**Figure 5:**
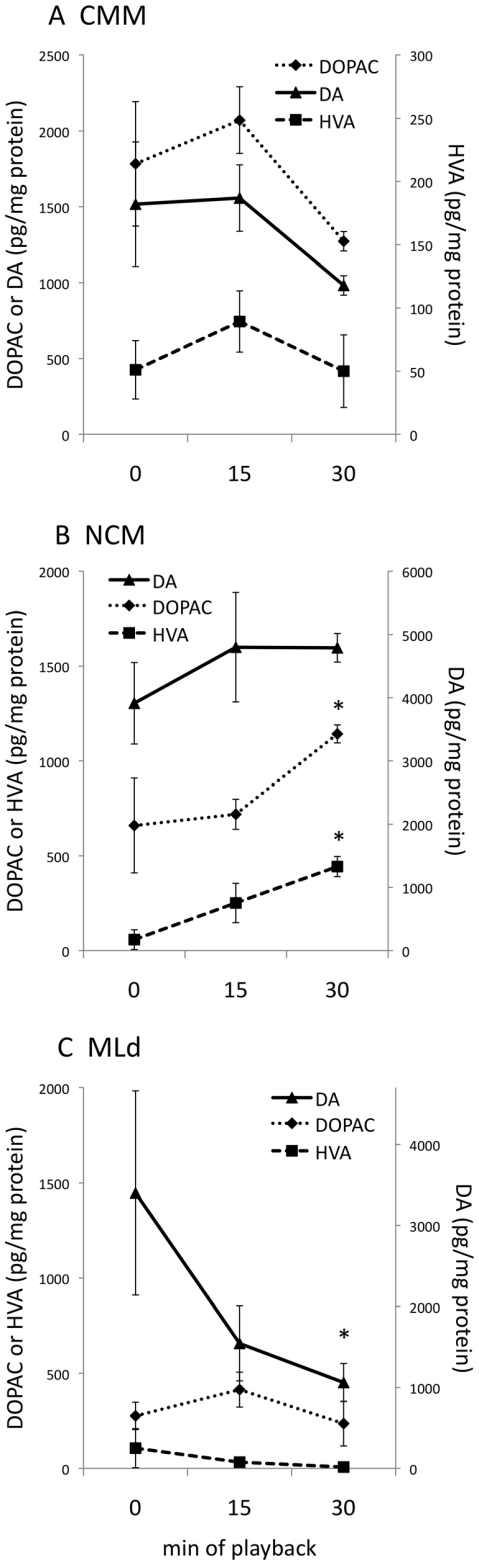
Hearing song alters catecholamine content in auditory areas. DA, dopamine. DOPAC, dihydroxyphenylacetic acid. HVA, homovanillic acid. A. CMM, caudomedial mesopallium. B. NCM, caudomedial nidopallium. C. MLd, dorsal portion of the lateral mesencephalic nucleus (auditory midbrain). *significantly different from silence (0 min song), see text for p values. n = 4 for each time point. Total protein content was available for all samples only for NCM (see Methods). In order to plot the values for CMM and MLd, we normalized them using the average protein values for those regions. Note the different scales on the Y axes in each graph.

In order to test whether the phosphorylation of TH occurred concomitantly with increases in catecholamine synthesis or turnover, we measured catecholamines and their metabolites *via* HPLC in a subset of the above females. Hearing song significantly increased the concentrations of two dopamine metabolites in NCM (Fig. 5B). The principal DA metabolite, dihydroxyphenylacetic acid (DOPAC), increased between 0 and 30 min of song exposure (z = 2.22, P = 0.026). For another metabolite, homovanillic acid (HVA), there was a trend at 15 min (z = 1.86, P = 0.063) and by 30 min this metabolite was also significantly elevated (z = 3.70, P<0.001). DA decreased significantly in MLd after 30 min (Fig. 5C; z = −14.03, P = 0.035). We did not find a significant effect of song exposure on NE or its metabolite 3-methoxy-4-hydroxyphenylglycol (MHPG) in any region of interest (data not shown).

### Lateralization

For the subset of brains subjected to both IHC and HPLC, we performed the two techniques on opposite hemispheres. Whether we used the left or the right for each technique was balanced in our design, so it was not necessary to include it in our statistical model. Due to recent evidence of lateralization in the auditory pathway of songbirds, however [19], [47]–[50] we ran the model first including hemisphere. We found no convincing effects or interactions; therefore, we removed hemisphere from the statistical model that we present below (see Methods). Removing hemisphere from the model did not affect any of the findings we report here.

### Explanatory Value of Vocalization Behavior

**Figure 6:**
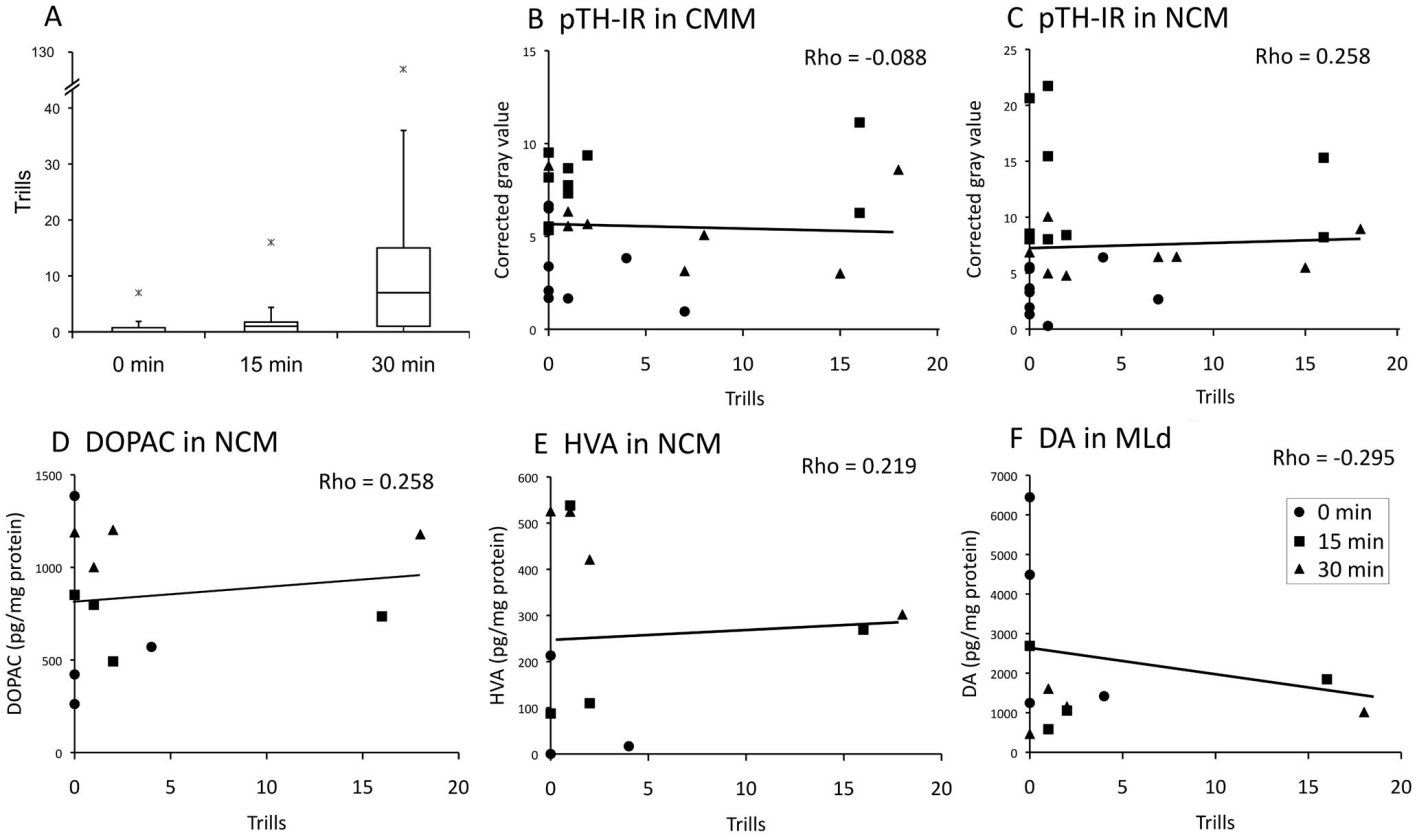
Significant effects of song playback (Figs. 3, 4) were not driven by vocal responses. A. Box-and-whisker plots showing the number of trills during the observation period. Outliers are marked by “X”. B–F, the number of trills did not explain levels of immunoreactivity for phosphorylated TH (pTH-IR), DA or DA metabolites in any region where a significant effect of playback was found. The trendlines shown in B and C include an observation, not shown on the graph, of a bird that trilled 126 times during the observation period.

In order to assess whether the birds’ own vocal responses may have increased CA activity, we recorded the behavior of each bird prior to tissue collection. The number of songs, tseets, or chip-ups (see Methods) did not vary according to playback duration (Kruskal-Wallis *K*≤0.602; P≥0.740; data not shown), suggesting that these behaviors were not driving the changes in catecholamine markers that we observed. The number of trills, which is part of the courtship response to song, was significantly higher in the birds that heard song (Kruskal-Wallis *K* = 7.388, P = 0.025; Fig. 6A). The number of trills was not, however, correlated with the catecholamine markers that were affected by song exposure (Spearman rho |R| ≤0.295; P≥0.177; Fig. 6B–F). It is therefore not the case that the birds with higher CA activity were the same birds that responded the most vocally to the playback. It is thus unlikely that the effects of hearing song on CA activity were caused by self-stimulation of the auditory pathway.

## Discussion

Our results are consistent with previous reports that social stimuli can induce CA activity in songbirds. In zebra finches, for example, presentation of song or other social stimuli induced FOS expression in TH-immunoreactive neurons in the brainstem [8], [27], [51]. In female European starlings, hearing high-quality song increased the density of noradrenergic fibers in the auditory forebrain over a period of one week [20]. In this study, we show that the induction of CA activity in the songbird auditory forebrain can be detected within 15 minutes of the onset of the sound. In sexually receptive female white-throated sparrows, hearing 15 or 30 minutes of conspecific male song increased the phosphorylation of TH, a rate-limiting enzyme in the CA synthetic pathway, in NCM and CMM of the auditory forebrain (Fig. 3). Because the phosphorylation of TH tightly regulates its activity [36], [52], we interpret this result to mean that hearing song induces catecholamine synthesis – either directly or in response to depletion. This activation was not sustained throughout an entire 30 min presentation of song, but rather was associated with the song onset (Fig. 3G). The subsequent dephosphorylation of TH may be due to depolarization-induced increases in intracellular calcium [53], [54]. Overall, our results suggest that catecholamines may do more than simply prime the auditory forebrain to respond to sound; rather, sound stimulation appears to be an important regulator of CA activity in the auditory forebrain.

Sound-induced CA activity has been described in the auditory system of rodents, in structures upstream from those we looked at in this study. In guinea pigs, for example, TH immunoreactivity was up-regulated in the cochlea after 24 hours of exposure to a 1 kHz tone [55]. In rats, exposure to 45 min of mildly intense white noise increased concentrations of the NE metabolite MHPG in the cochlear nuclei, but not the inferior colliculus or primary auditory cortex [56]. Our current findings provide evidence that sound exposure can induce CA activity in higher auditory centers and that this activity can be observed after only 15 minutes.

The detection of rapid increases in TH activity *via* IHC relies on the fact that in order to be fully active, TH must be phosphorylated at one or more serine sites [36], [52]. Of the possible sites, the best-understood is ser40, the phosphorylation of which is tightly regulated by stimulation *in vivo* [33], [34]. Ong and colleagues [54], [57] have shown that in rats, ser40 phosphorylation in the VTA and LoC increased in response to social but not non-social stressors. Riters et al. [38] used an antibody against TH phosphorylated at ser40 to assess CA responses to conspecific male song in female European starlings. They did not look at the auditory pathway in that study, but they did report changes in the hypothalamus and septum within 20 min after song onset. Because phosphorylation occurs within minutes of the application of a stimulus, immunolabeling of pTH may represent an accurate and convenient method for quantifying rapid changes in CA activity [32]. Because individual fibers can be visualized, this technique also allows precise neuroanatomical mapping of the effects. We are confident that the effects we report here are not due to increases in the availability of TH itself, because TH-IR was not altered in any region of interest after 15 or 30 min of sound exposure (Fig. 4). This result is consistent with other reports that the availability of TH protein is not affected rapidly by acute exposure to stimuli or pharmacological manipulation [36], [54], [57].

An over-arching principle of noradrenergic system organization is that axon collaterals can innervate sensory pathways at multiple levels. In other words a single LoC neuron may send axons to the auditory midbrain, thalamus, and forebrain so as to simultaneously influence auditory responses at all three levels [4]. In our study, we did not see evidence of such coordinated regulation, however. Whereas pTH-IR showed a clear peak at 15 min in the auditory forebrain, it was remarkably flat in Ov and MLd (Fig. 3). It is possible that the CA fibers innervating the auditory forebrain originate from sources largely different from those innervating lower structures in the pathway. Note that because TH is required for both DA and NE synthesis, dopaminergic and noradrenergic fibers alike were labeled in our material. A comparison of TH-IR with that of dopamine beta-hydroxylase (DBH), an enzyme specific to the NE pathway, shows that whereas DBH-IR fibers are far outnumbered by TH-IR fibers in the auditory forebrain, immunoreactivity for the two enzymes is roughly equal in MLd [19]. This pattern of labeling suggests that the relative contributions of DA and NE fibers to forebrain and lower auditory regions, respectively, may differ. Because the regulation of TH activity is accomplished *via* a wide variety of mechanisms, including phosphorylation at sites other than ser40 [34], we may not expect to see parallel effects in fibers originating from different sources.

We used the contralateral hemisphere of a subset of brains to test whether the changes in CA activity detected *via* IHC could also be detected *via* HPLC. Although our sample sizes were smaller for HPLC, we were able to detect a significant increase in the concentrations of two DA metabolites in NCM (Fig. 5A, C). This finding is consistent with sound-induced DA turnover in that area. It is possible that the increased concentration of metabolites indicates release followed by reuptake and degradation [58]–[61]. Alternatively, the rise in metabolites may indicate a surplus of newly synthesized DA that is broken down without being released [62]–[64]. In MLd, we noted a significant decline in DA that was not accompanied by an increase in either metabolite (Fig. 5C). Such a result could indicate DA release followed by metabolism to 3-methoxytyramine without reuptake, or a decrease in DA cell firing [58].

We hypothesized that the effects of song playback on CA activity measured *via* IHC would occur in parallel with those detected *via* HPLC. In NCM, this prediction was supported. We did not, however, obtain parallel results in CMM or MLd. Although pTH-IR was significantly enhanced in CMM after 15 min of song playback (Fig. 3G), we could not detect a concomitant change in the levels of catecholamines or their metabolites in CMM in the contralateral hemisphere (Fig. 5A). Further, although we did observe a significant decrease in DA in MLd (Fig. 5C), we found no effect of playback on pTH-IR in that region (Fig. 3I). There are a number of explanations for these disparate findings. First, the phosphorylation of TH and the synthesis and metabolism of catecholamines may occur at different times during a CA response. Although many researchers have reported effects of sensory stimulation on CA activity within 15 to 30 min [38], [54], [65], [66], we may have missed an effect that occurred earlier than 15 min or later than 30 min. Second, some authors have reported lateralization of function in the auditory pathway of songbirds [47]–[50]. If CA function is lateralized [19], we should not expect that CA activity will always be identical in both hemispheres. Although we did not find convincing evidence of lateralization in this study, our sample size for the HPLC assay was small. The small sample size, together with missing protein values for CMM and MLd (see Methods), may have contributed to our inability to detect changes *via* HPLC in those regions. Finally, as noted above, the phosphorylation of TH may occur at four possible serine sites [34]. Using our methods, we would not have been able to detect activation of TH *via* phosphorylation at a site other than ser40. The significant DA response, together with the lack of a pTH response, may suggest an alternate phosphorylation site in MLd fibers.

CA responses, in the form of NE or DA release, may occur *via* one or more distinct mechanisms such as tonic, phasic, or firing-independent release, the latter of which is mediated at the terminal [1], [21], [25]. We hypothesize that hearing song triggers phasic release, but it may also shift CA cells into a “high tonic” mode [67] or stimulate firing-independent processes that involve, for example, reverse transport of DA [68]. We cannot distinguish among these possibilities here. Our findings clearly suggest an increase in catecholamine synthesis, but although elevated levels of CA metabolite have been interpreted as evidence of release, they may also indicate breakdown without release [58]–[60], [62]–[64]. Playback studies using techniques that allow greater temporal resolution and unequivocal evidence of catecholamine release will be necessary to better understand the mechanisms underlying sound-dependent CA activity in the auditory forebrain.

The design of the present study raises two important questions that should be addressed in future work. First, does sound-induced CA activity depend on stimulus salience? Monoaminergic neuromodulators are thought to bring information about internal state and environmental context into sensory areas, thus helping to maximize responses to behaviorally relevant signals [1], [2], [69]. In this study, we played a courtship signal to receptive females, all of which likely found it highly relevant. We do not know whether the CA activity we observed would have been induced at a similar level by a less salient sound. Previous work in rats suggests that the CA activity induced by a sound may in fact depend on its behavioral relevance. Using microdialysis in the auditory cortex of rats, Stark and Scheich [61] showed that tone-induced HVA release was greatly enhanced while the rats were learning to associate the tones with a foot-shock. Similarly, in monkeys trained on a vigilance task, phasic CA discharges occur preferentially to target stimuli [4]. We hypothesize that sounds with high behavioral relevance, such as song, may induce more CA activity in the auditory forebrain than other sounds. Playback studies using more than one auditory stimulus will be necessary to test this hypothesis.

The second question that should be addressed is whether sound-induced CA activity depends on social context or reproductive state. The behavioral relevance of courtship signals waxes and wanes according to social context and the reproductive state of the listener. Perhaps as a consequence, the magnitude of auditory responses to those signals depends to some extent on the social environment and plasma reproductive hormones [70]–[72]. We have hypothesized that context- and estrogen-dependent auditory plasticity is accomplished *via* CA systems; exposure to high quality male song [20] and estradiol (E2) treatment [10], [19] independently increased the density of CA innervation of auditory areas. This enhanced innervation may prime auditory areas to respond more selectively to behaviorally relevant sounds. Here, we show that CA fibers innervating the auditory forebrain themselves respond rapidly to sound stimulation. A study wherein reproductive state or social context is manipulated would help to clarify the function of this response.

In addition to our finding that a biologically relevant sound induces CA activity, we recently showed evidence that the same is true of another monoamine, serotonin [46]. In E2-treated female white-throated sparrows, the concentration of the serotonin metabolite 5-hydroxyindoleacetic acid (5-HIAA) increased in NCM after 30 min of exposure to male conspecific song. Together, our findings suggest that serotonergic and dopaminergic activity are induced by the same stimulus on a similar time scale. Like catecholamines, serotonin is well-known to play a critical role in sensory plasticity by altering receptive fields, response thresholds, and signal-to-noise ratios in sensory areas [2]. In fact, although their mechanisms of action tend to differ within any particular sensory area, catecholamines and serotonin share function in that they alter the precision or selectiveness of auditory coding according to behavioral state [2], [73]. Our future work will involve receptor mapping and pharmacological manipulations to assess the likely functions of sound-induced monoamine activity in the auditory system of songbirds.

## Materials and Methods

### Ethics Statement

All procedures in this study were approved by the Emory University Institute for Animal Care and Use Committee and adhered to NIH standards.

### Animals

**Figure 7:**
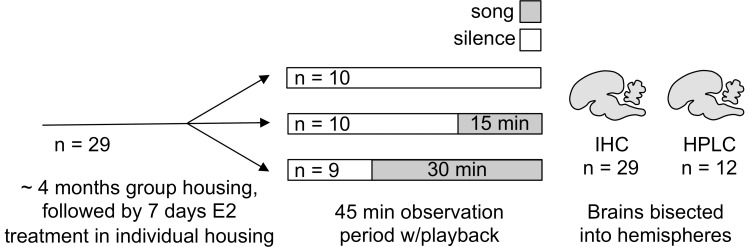
Time course of the experiment. Female white-throated sparrows were collected from a free-living population and housed on short photoperiod for approximately 4 months. Each then received a subcutaneous silastic implant filled with estradiol (E2) to mimic breeding levels. Seven days later, each was isolated in a sound-attenuating chamber and exposed to either silence, 15 min song, or 30 min song. Brains were harvested immediately after the observation period and bisected into hemispheres for analysis by immunohistochemistry (IHC) or high pressure liquid chromatography (HPLC). The hemisphere used for each procedure (left or right) was balanced across playback conditions.

The experimental design is depicted in Fig. 7. We collected a total of thirty female white-throated sparrows in mist nets in Atlanta, Georgia during fall 2007 and 2009. We determined their sex by polymerase chain reaction (PCR) analysis using a blood sample [74] and confirmed sex by necropsy at the end of the study. Prior to the study, the birds were housed at the Emory University animal care facility in indoor walk-in flight cages and supplied with food and water *ad libitum*. We held day length constant at 10∶14 h light-dark, which corresponds to the shortest day the birds would experience while overwintering at the capture site, for at least two months to ensure that they were not photorefractory [75], [76]. Seven days prior to the playback experiment, we transferred the birds in pairs to sound-attenuating chambers where they were housed individually in adjacent cages (38×38×42 cm).

### Estradiol Treatment

In captivity, female *Zonotrichia* sparrows do not undergo full ovarian recrudescence even under long day lengths [77]–[79]. We therefore treated the animals with exogenous E2 in order to simulate the hormonal milieu associated with breeding [28], [71], [72], [80]. On the day the birds were transferred to individual cages, we implanted each bird with a subcutaneous silastic capsule (length 12 mm, ID 1.47 mm, OD 1.96 mm, Dow Corning, Midland, MI) containing 17β-estradiol (Steraloids, Newport, RI) and sealed both ends with A-100S Type A medical adhesive (Factor 2, Lakeside, AZ). This dose of E2 increases plasma levels to those typical of the breeding season within seven days in this species [71], [81] and likely does so within two days [79].

### Song Playback

We started the playback experiment seven days after E2 treatment began. On the afternoon prior to playback, we isolated each female in a sound-attenuating chamber equipped with a speaker, a video camera, and a microphone. At ∼2 h after lights-on the following morning, we presented the song stimulus (see below) *via* the speaker inside the chamber. Each bird heard either 15 min of song (n = 10), 30 min of song (n = 9), or silence, i.e. no song stimuli (n = 10). Video and audio recordings were made of each bird before and during stimulus presentation. The chamber used for playback (one of three identical chambers) was balanced across playback duration.

An observer blind to the hypothesis quantified the vocalizations (chip-up calls, trills, tseets, and songs; see [81], [82] for descriptions) given by each female during the 45 min prior to tissue collection. This period consisted of 45 min of silence for the birds in the 0 min condition, 30 min of silence followed by the playback for birds in the 15 min condition, and 15 min of silence followed by the playback for birds in the 30 min condition (Fig. 7).

### Song Stimuli

The stimulus presentations have been previously described in detail [71], [72], [78], [81]. Briefly, we downloaded recordings of male white-throated sparrow songs from the Borror Laboratory of Bioacoustics birdsong database and constructed presentations consisting of one song every 15 s (natural song rate). To prevent habituation to the stimulus, the identity of the singer changed to a new male every three minutes. Thus, females listening to 15 min of song heard five unique males, and females listening to 30 min of song heard ten unique males. Within each group, each female heard the males in a unique order. All songs were presented at 70 dB, measured at the listener’s cage.

### Tissue Collection and Immunohistochemistry

Immediately after the stimulus presentation we rapidly decapitated each bird, quickly harvested the brain, and fixed it in 5% acrolein as described previously [80], [83]. We cut three series of 50 µm parasagittal sections from one hemisphere (left or right was balanced across groups) using a freezing sliding microtome and immunolabeled two of the three series using standard IHC protocols [10], [19], [38]. We incubated one of those series with an anti-pTH antibody (Genetex; Irvine, CA; see *antisera* below) diluted 1∶1250 [38], and the other with an anti-TH antibody (ImmunoStar; Hudson, WI; see *antisera* below) diluted 1∶2000 [10], [19]. We labeled the antigens in both series of sections using a biotinylated secondary antibody and the ABC method (Vector, Burlingame, CA). We visualized pTH immunolabeling with nickel-enhanced diaminobenzidine [84] and the TH immunolabeling using diaminobenzidine without nickel. We processed each series of brain sections in three separate runs of IHC in which the three playback conditions were balanced across runs. Following IHC, we mounted all of the sections onto gelatin-subbed microscope slides, dehydrated them, and coverslipped in DPX (Sigma, St. Louis, MO).

### Antisera

To label pTH we used a rabbit polyclonal antibody raised against a synthetic phosphopeptide corresponding to amino acid residues surrounding the phosphorylated ser40 of rat TH (Genetex, Cat#GTX16557). This antibody labels a CA-like distribution of cells and fibers similar to the distribution of TH cells and fibers in zebra finches, canaries, and white-throated sparrows [10], [19], [85], [86]. To validate the pTH antiserum, we followed Saper and Sawchenko [87]. Using tissue from two untreated females not in the study, we first determined the concentration at which labeling was barely discernable (1∶20,000) and then performed preadsorption tests at twice that concentration [87] and at the concentration normally used to label the protein (1∶1250). We incubated the diluted antibody with 50 µg/ml of TH phosphor S40 control peptide, supplied by the manufacturer (Genetex, Cat#GTX30707), with gentle agitation at least 3 hours at room temperature before use. Pre-adsorption completely abolished labeling of somata and fibers.

To label TH, we used a mouse monoclonal antibody generated against denatured TH purified from rat PC12 cells (ImmunoStar, Cat#22941). It immunolabels both unphosphorylated and phosphorylated TH. According to the manufacturer, the antibody recognizes a 62 kDa band corresponding to TH in rat, and does not cross-react with DBH, dihydropterdine reductase, phenyletholamine-N-methyltransferase, phenylalanine hydroxylase or tryptophan hydroxylase using Western blot methods. It has wide species cross-reactivity and has been validated by preadsorption studies in a range of vertebrates [88]. This antibody labels a catecholamine-typical pattern of neurons and fibers in a wide variety of birds [5], [85], [89]–[92] including white-throated sparrows [10], [19], [93] and was used by Reiner et al. [86] to perform an exhaustive characterization of the avian distribution of TH-immunoreactivity. Anti-TH antibodies from other sources and anti-DA antibodies produce the same neural distribution in birds [94], [95]. In our tissue, the antibody labels all major TH cell groups A1–A15 and fibers in a distribution typical of TH. We saw no specific labeling following omission of the primary or the secondary antibodies.

### Image Acquisition and Quantification of pTH and TH Immunolabeling

We conducted all image acquisition and analyses while blind to treatment group. To photograph each region of interest (ROI), we used the 10x objective on a Zeiss Axioskop microscope attached to a Leica DC500 camera and Macintosh G5 computer running Leica Firecam (version 1.7.1). We captured rectangular images (approximately 32 MB in size) corresponding to the field of view of the camera (870×690 µm), holding the light level constant for all photos. We based the exposure time and luminosity levels on those automatically set by the Firecam software.

We acquired images of NCM and CMM between ∼350 and ∼800 µm from the midline in the same four consecutive sections from each series. For CMM, the upper corners of the field of view of the camera were positioned along the dorsal boundary of CMM, one of the lower corners was positioned adjacent to the lamina mesopallium, and the entire photo was used in the analysis [19]. For NCM, we took photos with the rostrodorsal domain [72] positioned in the center, and used ImageJ (version 1.41o, National Institutes of Health, Bethesda, MD) to sample from a circular area approximately 550 µm in diameter. We did not attempt to sample Field L, first because there are very few TH fibers in this area [86], and second because the diffuse background staining we observed there was also present in sections with primary antibody omitted.

In addition to sampling the auditory forebrain, we also examined pTH and TH immunolabeling in the auditory thalamus (Ovoidalis, n. Ov) and midbrain (MLd). We photographed Ov in the three consecutive sections in which it was the largest [19] and then used ImageJ to trace the core and shell regions (Fig. 2G, I). We photographed MLd in the five consecutive sections in which it was the largest and traced the area corresponding to the core [96], also called the inner MLd [97], in ImageJ (Fig. 2 H, J). Finally, we quantified pTH and TH immunolabeling in a non-auditory region, HA, to test whether sound-induced phosphorylation of TH is specific to auditory regions. We took photos of HA at its rostral- and medial-most extent (just caudal to the olfactory bulb), and used the entire photo to quantify immunolabeling.

We converted all of the photos to 8-bit scale and calculated the average gray value of each ROI using ImageJ. We then calculated the average gray value of background labeling in each photograph by placing between 5 and 20 small circles into areas within or surrounding the ROI that did not contain immunoreactive fibers. We then subtracted the average gray value of the background samples in each photo from the average gray value of the ROI in that photo and took the absolute value of this difference, which we called “corrected gray value”. In a few cases, the background was slightly darker than the labeling in the ROI; in those cases we used “0”. The mean corrected gray value was then calculated for each ROI in each bird by averaging across sections.

### Statistical Analysis of IHC Data

To analyze the effects of song exposure pTH-IR, we used a separate general linear model (Stata) for each ROI. These models use restricted maximum likelihood to estimate parameter coefficients and z tests to determine whether the value of a coefficient differs from 0. For each model, the response variable was the mean corrected gray value for pTH-IR (see above). The predictor, song duration, was expanded into a dummy-variable set to model the contrast between 0 and 15 min and the independent contrast between 0 and 30 min. Because increases in pTH-IR could be due to increases in the availability of TH rather than an increase in phosphorylation, the same analyses were also performed for TH-IR.

### Measurement of Catecholamines and their Metabolites in Regions of Interest

The majority of the material used to label pTH and TH was obtained from birds collected in 2007 (n = 5 or 6 in each playback condition). In order to test whether hearing song had rapid effects on the actual levels of catecholamines and their metabolites, we wanted to quantify these compounds in brain tissue using HPLC. We therefore added 12 females to the study in 2009, using methods identical to those outlined above. Of these 12 birds, four heard silence, four heard 15 min of song, and four heard 30 min. After the brains were collected, we used a clean razor blade to bisect each brain into hemispheres. We fixed one in 5% acrolein for IHC as described above, thus increasing the sample size for the IHC portion of the study to n = 10 in the 0 and 15 min conditions, and n = 9 in the 30 min condition. We flash-froze the other hemisphere to be shipped to the University of North Carolina for HPLC analysis (see below). The hemisphere that was flash-frozen (right or left) was balanced across playback duration.

We determined the concentration of catecholamines and metabolites by HPLC with electrochemical detection [98] using micropunches of the ROI in the flash-frozen hemispheres. We considered n. Ov too small to sample accurately *via* micropunch, so our samples were limited to CMM, NCM, and MLd. The methods for quantification of catecholamines and metabolites are published elsewhere [20], and we reiterate the relevant portions here. We sectioned the frozen, non-fixed hemispheres at −12°C in the sagittal plane at 300 µm on a cryostat, thaw mounted the sections onto glass slides, and rapidly re-froze them on dry ice. From each of two consecutive sections and using chilled thin-walled stainless steel spring-loaded punch tools (Fine Science Tools, Foster City, CA, USA), we micropunched a region of CMM (0.5 mm i.d.) and of NCM (1 mm i.d.), each for which the anatomical boundaries have been described [70]. We also took a micropunch (0.5 mm i.d.) from a region of MLd from two consecutive sections. We expelled the tissue punches into 1.9 ml polypropylene microcentrifuge tubes (one for each punch), froze them on dry ice and stored them at −80°C until assay.

The mobile phase consisted of sodium acetate (3.1 g), monohydrate citric acid (8.84 g), disodium EDTA (5 mg), sodium octyl sulfonate (215 mg), HPLC grade methanol (200 ml) and double-distilled, deionized water (800 mL). Immediately before the assay, we added 125 µL of mobile phase with a concentration of 1 pg/µL of the internal standard, isoproterenol (Sigma), to each tube. We sonicated the samples and then centrifuged them at 16,000 *g* for 15 minutes at 4°C. We aspirated the supernatant and injected 100 µl from each sample into the HPLC system.

The chromatographic system consisted of a HTEC-500 HPLC machine (EICOM Corporation, Kyoto, Japan), MIDAS Autosampler (Spark Holland, Emmen, Netherlands) and EPC-500 PowerChrom software Version 2.5 (EICOM Corporation, Kyoto, Japan) running on a PC. A precolumn (PC-04, 100×3.0 mm i.d., EICOM Corporation, Kyoto, Japan) was applied to the system to avoid contamination of the separation column (EICOMPAK SC-30DS, EICOM Corporation, Kyoto, Japan). We separated the compounds with mobile phase and the flow rate was 350 µl/min. We maintained the electrode potential at 750 mV with respect to an Ag/AgCI reference electrode. We prepared standard solutions containing either 10 or 1 pg/µl of the five external standards (Sigma): DA, NE, DOPAC, HVA, and MHPG, and the internal standard, isoproterenol. The higher concentration of external standards produced peaks with areas ten times greater than did the lower concentration (ratio 10.5+/−0.5 SD) showing that the standards were reliably detected. To calculate the amount of catecholamines and metabolites in the samples, we compared the area of each compound peak with the area of their corresponding external standards.

We measured the protein content of each sample by dissolving the remaining sample pellet in 0.2 N NaOH (100 µl) and performing the Bradford protein-dye binding assay (Quick Start Bradford Protein Assay, Bio-Rad) with bovine serum albumin as a standard (Bio-Rad) on a µQuant microplate spectrophotometer (BioTek) [99].

### Statistical Analysis of HPLC Data

In some cases, we had difficulty getting reliable protein measurements. As a result, we had a complete set of protein measurements only for NCM; for CMM and MLd we were missing the protein measurement for at least one sample. We therefore normalized for protein content only for NCM. The data from CMM and MLd were not normalized. To accommodate this discrepancy, we compared the concentrations of each compound only between playback conditions and not ROIs. We used a separate general linear model for each compound and each ROI as described for pTH above. For each model, the response variable was the mean concentration from the two punches for that ROI (in pg/mg protein for NCM and pg for CMM and MLd; see above), and the predictor song duration was expanded into a dummy-variable set to model the contrast between 0 and 15 minutes and the independent contrast between 0 and 30 minutes.

### Statistical Analysis of Behavioral Data

We used Kruskal-Wallis ANOVAs to test whether playback duration affected vocalization behavior, followed by Spearman correlation tests to rule out the birds’ own vocalizations as a source of variability for the CA variables that were affected by playback duration.
